# OP07 Technical Aspects Of Artificial-Intelligence-Based Tools Applied In Health Technology Assessment Processes: A Scoping Review

**DOI:** 10.1017/S0266462324000710

**Published:** 2025-01-07

**Authors:** Denis Satoshi Komoda, Marilia Mastrocolla de Almeida Cardoso, Ana Renata Lima, Marília Berlofa Visacri, Carlos Roberto Silveira Correa, Brígida Dias Fernandes

## Abstract

**Introduction:**

In the last decade, artificial intelligence (AI) has been increasingly applied in health technology assessment (HTA) to accelerate evidence synthesis, optimize resources allocation, and guarantee timely delivery of trustworthy technologies in health. The aim of the present scoping review is to map AI models applied in HTA, and technical characteristics of AI-based automation and semi-automation applied in HTA.

**Methods:**

A search strategy containing core expressions “AI” and “HTA” and correlated terms was conducted in nine specialized databases (health and informatics) in February 2022. Inclusion criteria were publications testing AI models applied in HTA. Study selection was performed by independent pairs, with consensus meetings. No filters were applied. Data on year and country of publication, HTA phase, subsets of AI (e.g., machine learning [ML], neural networks), type of algorithm (e.g., support vector machine [SVM], K-nearest neighbors), and performance scale were extracted. Data were analyzed as descriptive frequency statistics. Used metrics will be presented narratively.

**Results:**

Sixty-one publications were included. The first study identified was published in 2006, and since then the number of publications has been consistently growing, with 11 publications in the year 2021. Canada, USA, and the UK concentrate 72 percent of publications (44 in 61) equally distributed. The most common HTA phase was the evidence synthesis, with 59 studies (96%). The main task performed was study screening/selection (66.6%). The majority of ML models (80.9%) contained two learning nodes or fewer, and applied SVM and decision-tree-based algorithms. Inter-rater agreement, accuracy, and 95 percent recall were the most common scales observed.

**Conclusions:**

Although recent developments in AI applied to HTA show increasing potentiality, studies are concentrated in the study selection phase of evidence synthesis. Many areas need further development, such as horizon scanning and policymaking processes. Additionally, studies reporting time gain and economic gain outcomes are scarce and should be considered for the development of future studies in the field.

